# Association of Risperidone With Gastric Cancer: Triangulation Method From Cell Study, Animal Study, and Cohort Study

**DOI:** 10.3389/fphar.2022.846455

**Published:** 2022-04-04

**Authors:** Vincent Chin-Hung Chen, Tsai-Ching Hsu, Chiao-Fan Lin, Jing-Yu Huang, Yi-Lung Chen, Bor-Show Tzang, Roger S. McIntyre

**Affiliations:** ^1^ Department of Psychiatry, School of Medicine, Chang Gung University, Taoyuan, Taiwan; ^2^ Department of Psychiatry, Chang Gung Medical Foundation, Chiayi Chang Gung Memorial Hospital, Chiayi, Taiwan; ^3^ Institute of Medicine, Chung Shan Medical University, Taichung, Taiwan; ^4^ Clinical Laboratory, Chung Shan Medical University Hospital, Taichung, Taiwan; ^5^ Immunology Research Center, Chung Shan Medical University, Taichung , Taiwan; ^6^ Department of Child Psychiatry, Linkou Chang Gung Memorial Hospital, Taoyuan, Taiwan; ^7^ Department of Healthcare Administration, Asia University, Taichung, Taiwan; ^8^ Department of Psychology, Asia University, Taichung, Taiwan; ^9^ Department of Biochemistry, School of Medicine, Chung Shan Medical University, Taichung, Taiwan; ^10^ Mood Disorders Psychopharmacology Unit, University Health Network, University of Toronto, Toronto, ON, Canada; ^11^ Department of Psychiatry, University of Toronto, Toronto, ON, Canada

**Keywords:** risperidone, gastric cancer, cell study, animal study, cohort study, depression, bipolar disorder

## Abstract

**Purpose:** To examine the effects of risperidone, an atypical antipsychotic agent, on gastric cancer.

**Methods:** A triangulation method comprising bench studies, including cell and animal experiments, and a retrospective cohort study, was subsequently performed.

**Results:** The bench study indicated that risperidone exerted more prominent tumor inhibition effects than other atypical antipsychotics on the proliferation of KATO-III cells, a human gastric cancer cell line. Significant and dose-dependent cell viability was observed in Hs27 cells (control cells) in the presence of risperidone compared with in KATO-III cells. Both *in vivo* and *in vitro* results indicated that risperidone significantly inhibited the proliferation of KATO-III cells by inducing ROS and apoptosis, and that it suppressed the growth of xenografted KATO-III tumors in nude mice. In addition, the population-based cohort study found that risperidone users had reduced risks of gastric cancer compared with non-users, with lowered adjusted hazard ratios (HRs) for two induction periods (HR = 0.75; 95% confidence interval [CI] 0.68–0.83 for the one-year induction period, and HR = 0.68; 95% CI 0.61–0.75 for the two-year induction period).

**Conclusion:** The findings are consistent with anticancer effects associated with risperidone, but further research and evaluations are warranted.

## Introduction

Gastric cancer (GC) is the third most common cause of cancer mortality in men and the fifth most common cause in women, resulting in approximately 740,000 deaths worldwide every year (Jemal et al., 2011). Evidence has indicated that Eastern Asia exhibits the highest rate of GC incidence (Colquhoun et al., 2015; Ashrafizadeh et al., 2020a). Surgery is currently the most common treatment for localized GC at early stages (Ashrafizadeh et al., 2020b). However, early symptoms of GC are often non-specific, and delayed diagnosis frequently results in a poor prognosis, with 5-year survival rates of less than 40% reported (Sisic et al., 2018). Notably, GC malignant cells exhibit metastatic nature that invades neighboring and distant tissues such as lung, bone and liver, making it difficult to completely eradicate cancer cells (Ashrafizadeh et al., 2020a). The limited treatment response indicates that the development of novel and safe therapeutic agents is warranted. Hence, increased attention has been focused on understanding the underlying mechanisms of GC and developing the alternative remedies for GC malignancy (Abadi et al., 2021).

The potential beneficial effects of antipsychotic agents on cancer risk have attracted interest, because epidemiological studies have indicated that patients with schizophrenia exhibit a lower than expected incidence of cancer (Grinshpoon et al., 2005; Chou et al., 2011). Antipsychotics have been proposed to play a role in this phenomenon, with several *in vitro* studies supporting antitumor effects (Cheng et al., 2015; Lu et al., 2015; Yin et al., 2015). However, some preclinical and drug screening studies have provided contradictory evidence, wherein several antipsychotics are associated with carcinogenicity (Brambilla et al., 2009; Amerio et al., 2015), and a review concluded contradictory results from laboratory experiments, with the former suggesting anticancer attribute while rodent studies have suggested that some antipsychotics, including risperidone, increase the risks of certain cancers (Fond et al., 2012).

Epidemiological studies have also provided inconsistent findings. Some have indicated null or negative relationships between antipsychotic use and the risks of breast cancer, colon cancer, and prostate cancer (Dalton et al., 2006) as well as a decreased risk of rectal cancer (Dalton et al., 2006). Others have found antipsychotic use to be associated with higher risks of leukemia (Nielsen and Boysen, 2010) and ovarian cancer (Harlow et al., 1998). Furthermore, some epidemiological findings have been unreproducible in bench studies: for example, the anticancer activity of olanzapine on hepatocellular carcinoma (Chen et al., 2019).

Studies on the beneficial or risk effects of antipsychotic agents on gastric cancer are scant. One population-based case-control study found that antipsychotics, including second-generation agents, were associated with reduced risk of gastric cancer (Hsieh et al., 2019). However, a cell study found antitumor effects associated with the first-generation antipsychotic thioridazine (Mu et al., 2014), while another study found a similar association with the second-generation antipsychotic sertindole (Dai et al., 2020).

## Materials and Methods

The method of this study was based on findings obtained from our population-based case–control study mentioned above (Hsieh et al., 2019), in which showed a link between antipsychotics and a lower risk of gastric cancer. Clozapine, flupentixol, quetiapine, and risperidone were selected for this study and their influences on cell viability were explored. Cell viability was detected according to our aforementioned report (Chen et al., 2018). The 3-(4,5-cimethylthiazol-2-yl)-2,5-diphenyl tetrazolium bromide (MTT) test was adopted to screen the effects of clozapine, flupentixol, quetiapine, and risperidone on the survival of SW480 cells. Notably, the viability of KATO-III cells was lower after treatment with risperidone compared with after treatment with clozapine, flupentixol, and quetiapine. Because risperidone revealed the most toxic consequent on KATO-III cells, we performed further bench experiments and population-based cohort study using only risperidone. The IACUC and IRBs of Chang Gung Medical Foundation approved this study (IACUC and IRB certificate approval number: 2015040102 and 201700253B0C501).

## Bench Study

### Cells and Antipsychotic Drugs

A human gastric carcinoma cell line (KATO-III [ATCC® HTB-103™]) and a human normal foreskin fibroblast cell line (Hs27 [ATCC ® CRL-1634™]) were purchased from ATCC and cultured in RPMI 1640 Medium supplemented with 20% fetal bovine serum (FBS) or in Dulbecco’s Modified Eagle’s Medium supplemented with 10% FBS, respectively. Antipsychotic drugs, including clozapine, flupentixol, quetiapine, and risperidone, were obtained from the Chiayi Chang Gung Memorial Hospital.

### MTT Assay

For determining cell viability, an MTT assay was performed. Briefly, cells were cultured in a 96-well plate (5 × 10^3^ cells per well) overnight at 37°C in an incubator. The medium in each well of the 96-well culture plate was replaced with fresh medium containing various concentrations of antipsychotic drugs for 24- or 48-h incubation. The medium was next replaced with 0.2 ml of MTT reagent (0.5 mg/ml) and reacted for another 4 h, and 0.2 ml of dimethyl sulfoxide was then mixed. Absorbance was detected at 570 nm using a microplate reader (EnSpire Series Multilabel Plate Readers), and viability of the cells was considered as the ratio of sample’s absorbance relative to the control’s absorbance.

### Flow Cytometry Analysis

To detect the proportions of different cell cycle stages for KATO-III cells in the presence of risperidone, flow cytometry analysis was conducted. Cells were incubated with different doses of risperidone for 1 day and then fixed with 70% alcohol for 12–16 h at 4°C after washing with phosphate buffered saline (PBS). Ten microliters of propidium iodide solution was then added, and the mixture was chilled in ice in the dark. The stained cells were analyzed using an FACSCanto II flow cytometer (BD Biosciences, USA) after filtration through a 40-μm nylon screen.

### Annexin V Assay

Annexin V assay was performed to detect apoptosis in KATO-III cells in the presence of risperidone. Briefly, KATO-III cells were cultured in the presence of risperidone for a day. After collection of cells through centrifugation, 0.1 ml annexin-binding buffer containing 1% annexin V-Fluorescein isothiocyanate (FITC) was then added. Subsequently, annexin V-FITC (5 µl) and of propidium iodide (1 µl) were added, and the mixtures were stand in the dark at 25°C for 20 min. Cells were immediately examined with a FACSCanto II flow cytometer (BD Biosciences).

### Xenograft Study

Fifteen 5-week-old male athymic nude mice (BALB/c nude mice) were obtained from the National Center for Experimental Animals (National Science Council, Taiwan) and maintained in a specific-pathogen-free facility at 22°C under 12-h light/dark cycles. All agreements were authorized by the Institutional Animal Care and Use Committee of Chiayi Chang Gung Memorial Hospital, Taiwan (IACUC#: 2015040102). KATO-III cells were infused subcutaneously into the flanks of 6-week-old mice. The doses of risperidone used in this study were 0.25 mg/kg or 1 mg/kg according to a previous study (Dilly et al., 2017). When the tumor volume was about 20 mm^3^, mice were stochastically separated into the following groups by daily administrating with (Jemal et al., 2011) PBS (Colquhoun et al., 2015), 0.25 mg/kg, and (Ashrafizadeh et al., 2020a) 1 mg/kg risperidone by oral gavage for 5 weeks, respectively. Xenograft tumor volumes were calculated every week through measurement of the diameters of tumors using a caliper. Euthanasia of mice was performed at the end of experiments by inhalation of carbon dioxide and the xenograft tumors were excised.

### Statistics

Statistical analyses were performed using GraphPad Prism 5 software (GraphPad Software, La Jolla, CA, United States) with one-way analysis of variance (one-way ANOVA). Tukey’s multiple comparisons test was then performed to determine the significance level. Three repeated experiments were performed, and all data are represented as standard error of the mean. *p* < 0.05 was considered statistically significant.

## Population-Based Study

### Data Source

The data used in this nationwide cohort study were obtained from the Taiwan National Health Insurance Research Database (NHIRD), which is maintained by the National Health Research Institute (NHRI). The database includes information on outpatient, ambulatory and hospital inpatient care services provided since 1 March 1995. The insurance program covers 99.5% of the national population (Ng, 1997). The database contains comprehensive data, including patients’ demographic data, dates of clinic visits and hospitalizations, disease diagnosis codes, procedure codes, and details of prescriptions. A validation study has demonstrated that the dataset exhibits excellent sensitivity and specificity for cancer diagnoses (sensitivity: 91.5% and specificity: 93.6) in the NHIRD (Kao et al., 2018).

### Exposure Assessment

For this study, we used the NHIRD and identified 29,548,231 participants who received at least one inpatient diagnosis for any psychiatric disorder (International Classification of Disease, Ninth Revision [ICD-9] codes: 290–319) or more than two outpatient diagnoses within 1 year between 1 January 2000, and 31 December 2013. We identified 233,216 participants who were prescribed risperidone (Anatomical Therapeutic Chemical [ATC]: N05AX08) from 2000 to 2010. Risperidone non-users were selected from the Longitudinal Health Insurance Database 2005 (LHID 2005), containing 1,000,000 participants randomly sampled from the year 2005 Registry for Beneficiaries of the NHIRD. The distribution of sex and age of the sampled participants in the LHID2005 did not significantly differ from that of the general population. After the exclusion of risperidone users, 989,000 participants remained in the LHID2005 from 2000 to 2010.

### Assessment of Main Outcomes

The main outcome of this study was gastric cancer (ICD-9 code: 151). Participants with a diagnosis of gastric cancer before the prescription of risperidone were excluded. Participants were followed up for the incidence of gastric cancer, as the study outcome, to the end of 2013.

### Covariates

Several covariates were selected, including sex, age, comorbid medical disorders, and concomitant medication use. Comorbid medical disorders were classified into physical comorbidities and mental comorbidities. For physical comorbid medical disorders, hypertension, hyperlipidemia, diabetes, chronic obstructive pulmonary disease, chronic kidney disease, peptic ulcers, and liver cirrhosis were examined. For mental comorbid medical disorders, major depressive disorder, bipolar disorder, schizophrenia, and alcohol use disorder were screened. Concomitant medication use included the use of aspirin, nonsteroidal anti‐inflammatory drugs, and statins. The covariate statuses in participants were examined during the follow-up period from 2000 to 2013.

### Statistical Methods

The descriptive results are presented as frequencies and percentages for categorical variables and as means with standard deviations and medians with interquartile ranges for continuous variables. We defined two induction periods for risperidone (i.e., one-year and two-year periods) to explore the effects of risperidone on gastric cancer risk. We conducted a time-dependent Cox proportional hazard model to explore the risk of gastric cancer in risperidone users compared with nonusers for these potential induction periods with SAS Version 9.4 (SAS Institute, Cary, NC) because we considered risperidone use as a time-varying variable.

The index date was set as 1 January 2000. and in the time-dependent Cox proportional hazard model, two observations were recorded for each risperidone user. Their first observation was considered as non-use of risperidone in the time between the index date and the first use of risperidone and the addition of the induction period. Their second observation was during the remaining time of follow-up to the event (i.e., gastric cancer development) or censoring (i.e., by 31 December 2013). However, if events occurred within the induction period, the second observation was unnecessary and therefore deleted, and the total follow-up times for these individuals were recorded based on their first observation of nonuse from the index date to the date of the event. By contrast, only one observation was recorded for risperidone non-users, with their total follow-up periods from the index date to the event or censoring. A multivariable time-dependent Cox proportional hazard model was used to identify potential risk factors with adjustment for demographics and physical and mental comorbidity and drug use.

Finally, we conducted a subgroup analysis to focus on middle- and old-age participants because gastric cancer onset is common in this age group. In this subgroup analysis, we included only participants 40 years or older on 1 January 2000, the beginning date of the follow-up. Hazard ratios (HRs) and 95% confidence intervals (CIs) were estimated. All analyses were two-tailed and unpaired, with significance set at *p* < 0.05.

## Results

### Bench Studies

#### Effects of Antipsychotics on Proliferation of Gastric Cancer Cells

**FIGURE 1:**
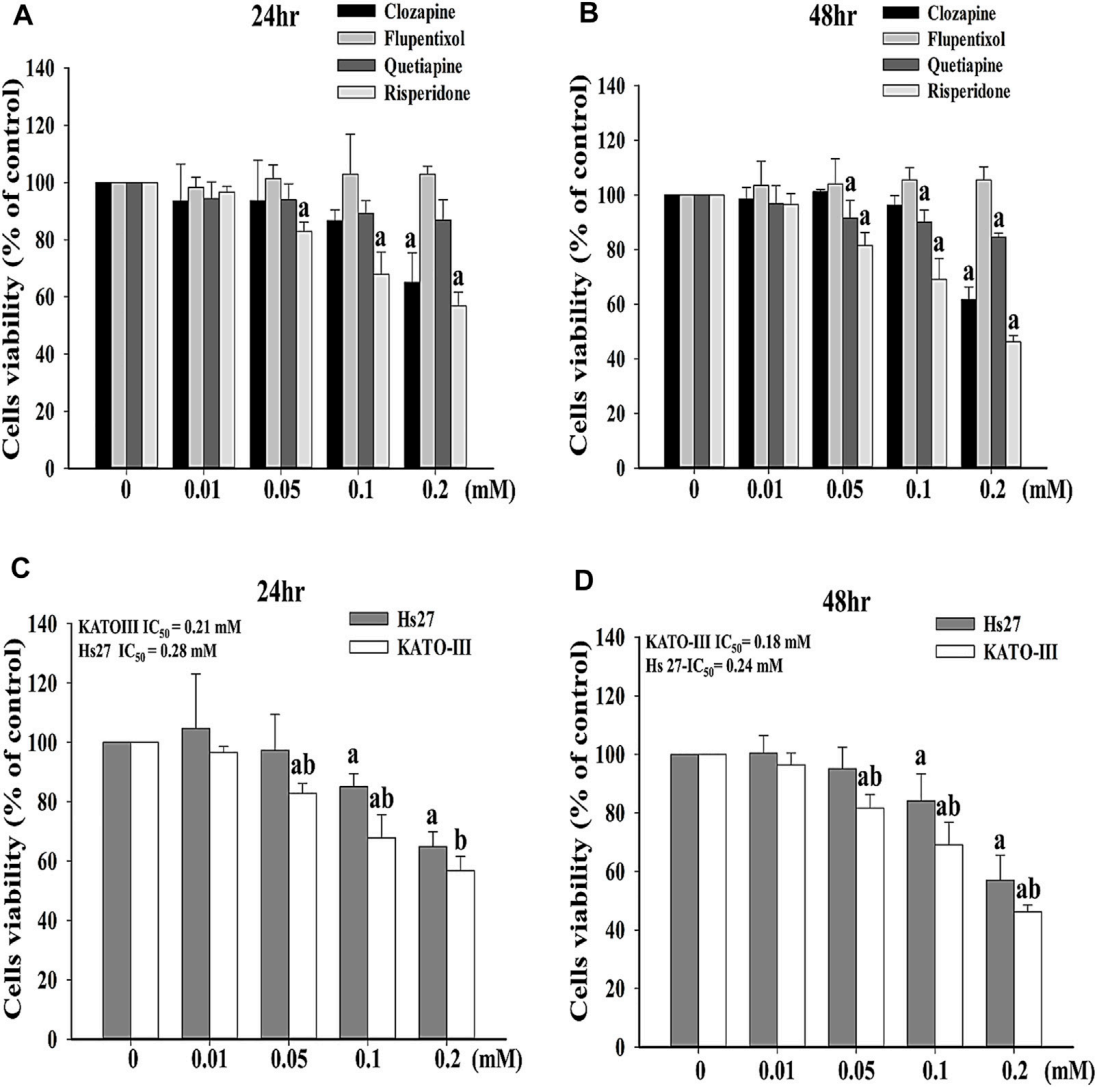
Effects of different antipsychotics on survival of KATO-III cells. Cell viability of KATO-III cell in the presence of clozapine, flupentixol, quetiapine, and risperidone for **(A)** 24 and **(B)** 48 h. Cell viability of KATO-III and Hs27 cells in the presence of risperidone for **(C)** 24 and **(D)** 48 h. Three-repeated tests were completed. The letters a and b indicate a significant difference (*p* < 0.05) compared with control (0 mM) or Hs27, respectively.

To investigate the effects of antipsychotics, including clozapine, flupentixol, quetiapine, and risperidone, on cell survivability, MTT assay was adopted. Notably, significantly lower viability for KATO-III cells was observed after treatment with 0.05, 0.1, and 0.2 mM risperidone for 24 h or 48 h compared with after treatment with clozapine, flupentixol, or quetiapine, respectively (Figures 1A,B). Since risperidone showed the most cytotoxic influence on KATO-III cell proliferation, we performed further analysis using only risperidone. The cytotoxic activity of risperidone was compared between KATO-III and Hs27 cells, a normal human foreskin fibroblast cell line. Notably, obviously survival of Hs27 cells was detected in the presence of risperidone compared with KATO-III cells (Figures 1C,D).

#### Risperidone Induces Apoptosis in KATO-III Cells via Increasing ROS Generation

**FIGURE 2:**
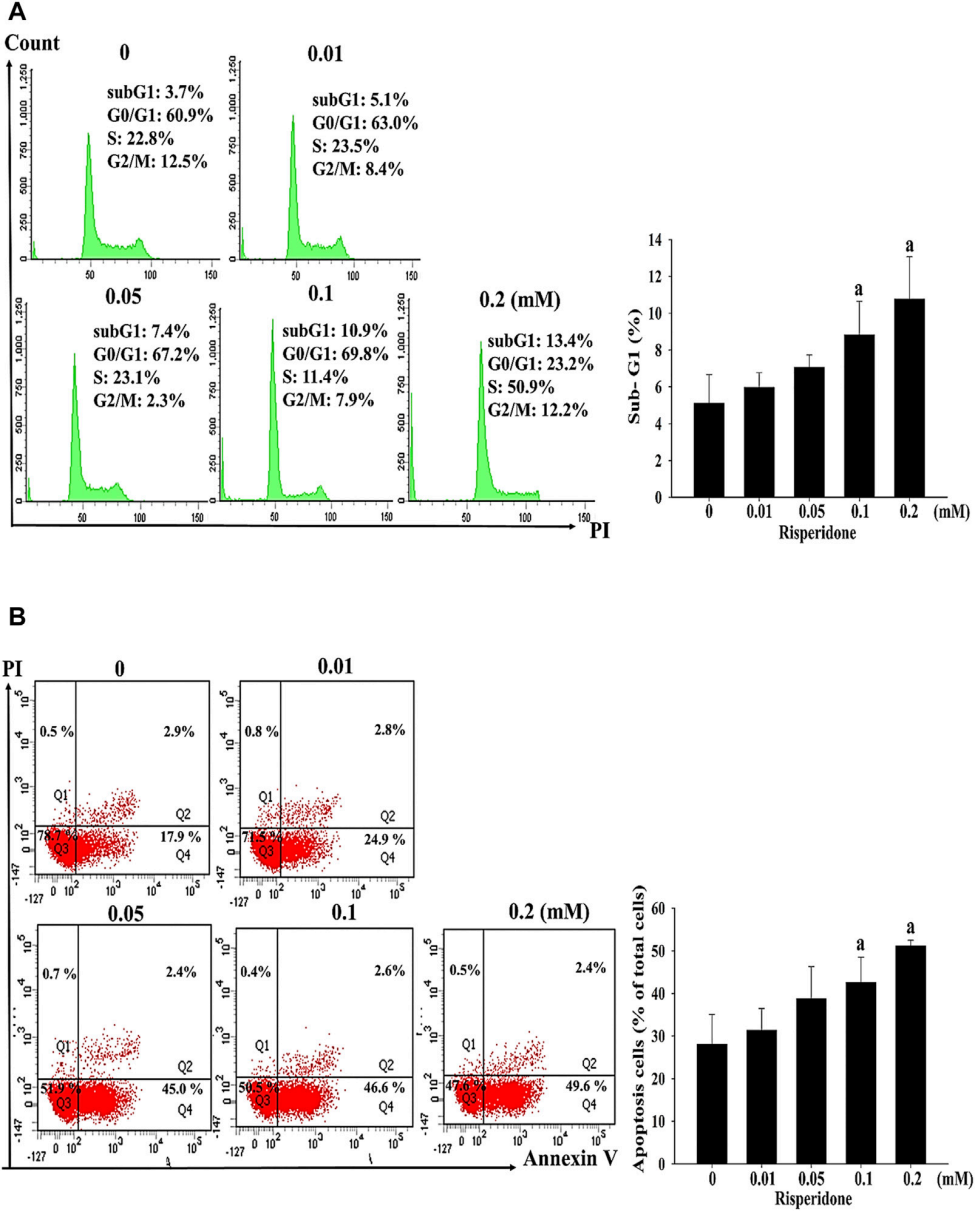
Risperidone increases sub-G1 proportion and apoptosis in KATO-III cells. KATO-III cells were treated with different concentrations of risperidone for 24 h. **(A)** Representative histogram results of flow cytometry analysis. Bar diagram showing the percentage of sub-G1 proportion. **(B)** Representative results of Annexin V. Bar diagram showing the percentage of apoptotic cells. Three-repeated tests were completed. The letter a indicates a significant difference (*p* < 0.05) compared with control (0 mM).

**FIGURE 3:**
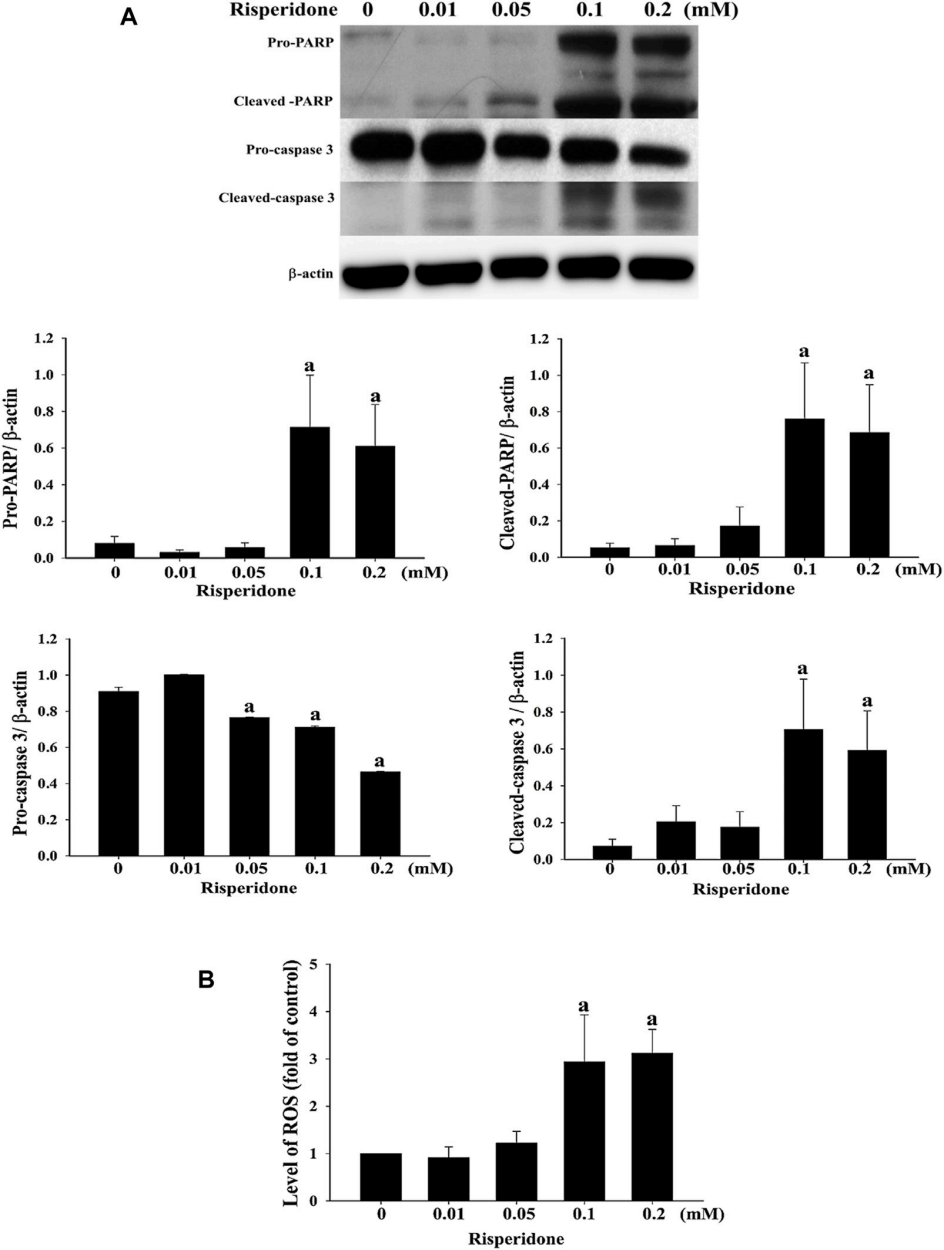
Risperidone increases the expressions of apoptotic proteins and ROS. KATO-III cells were treated with different concentrations of risperidone for 24 h. **(A)** Expressions of PARP, cleaved-PARP, pro-caspase 3 and cleaved-caspase 3 were detected with immunoblotting. **(B)** Level of ROS. Bars present the ratio on the basis of β-actin. Three-repeated tests were completed. The letter a indicates a significant difference (*p* < 0.05) compared with control (0 mM).

To verify whether apoptosis is inivolved in risperidone-induced cell death in KATO-III cells, flow cytometry and annexin V analysis were performed. Accordingly, a significantly higher sub-G1 proportion and annexin V signal were measured in KATO-III cells receiving risperidone in a dose-dependent manner (Figures 2A,B). Expressions of PARP and caspase-3 proteins were further examined to confirm the involvement of apoptosis in risperionde-induced KATOT-III cells death. Significantly increased amounts of cleaved-PARP and caspase-3 were detected in KATO-III cell in the presence of 0.1 and 0.02 mM risperidone (Figure 3A). Moreover, significantly increased levels of ROS were observed in KATO-III cells receiving 0.1 and 0.02 mM risperidone, respectively (Figure 3B).

#### Risperidone Inhibits the Growth of Xenograft KATO-III Tumor *In Vivo*

**FIGURE 4:**
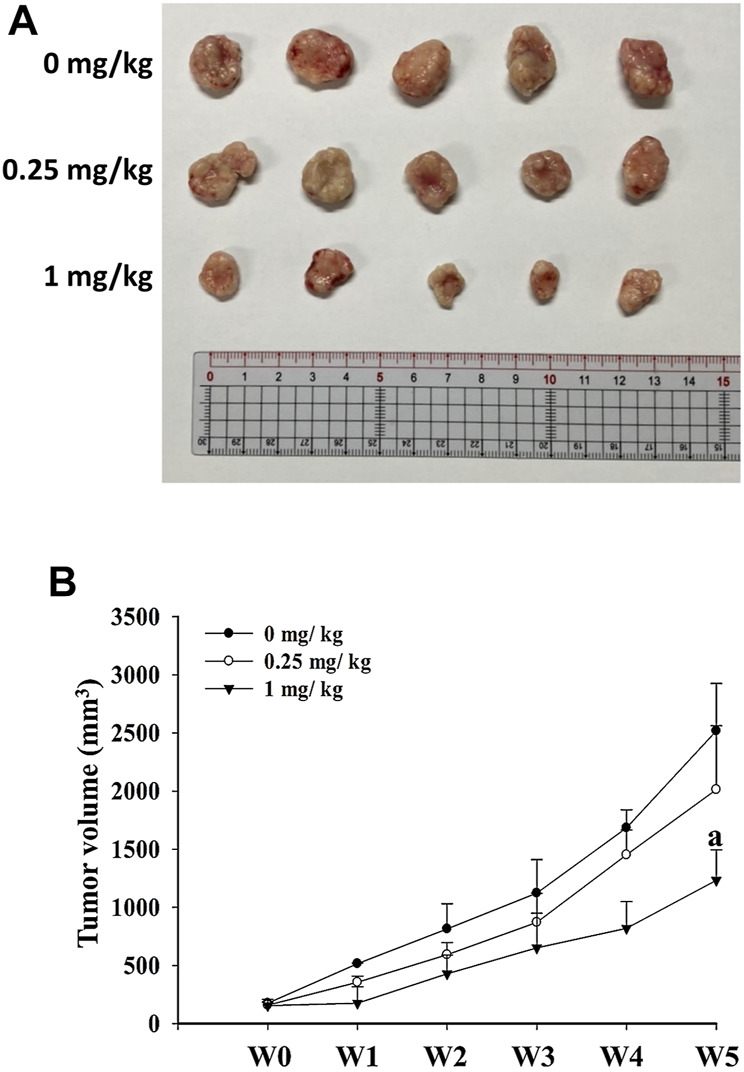
Risperidone inhibits the growth of xenograft KATO-III tumor. Athymic nude mice were divided into three groups (*n* = 5 per group) receiving 0, 0.25, and 1 mg/kg risperidone, respectively. **(A)** Representative images of excised xenograft tumors from different groups of mice. **(B)** Line graph shows the xenograft tumor volumes of mice from different grouips in different weeks. The letter a indicates a significant difference (*p* < 0.05) compared with control (0 mg/kg).

To verify the effects of risperidone treatment *in vivo*, xenograft KATO-III tumor was generated in BALB/c nude mice. Representative images of excised xenograft tumors were shown in Figure 4A. Notably, significantly lower mean tumor volume was detected in mice that were administered with 1 mg/kg of risperidone than control mice toward the end of the experiments (Figure 4B).

### Population-Based Study

#### Sample Characteristics

**FIGURE 5:**
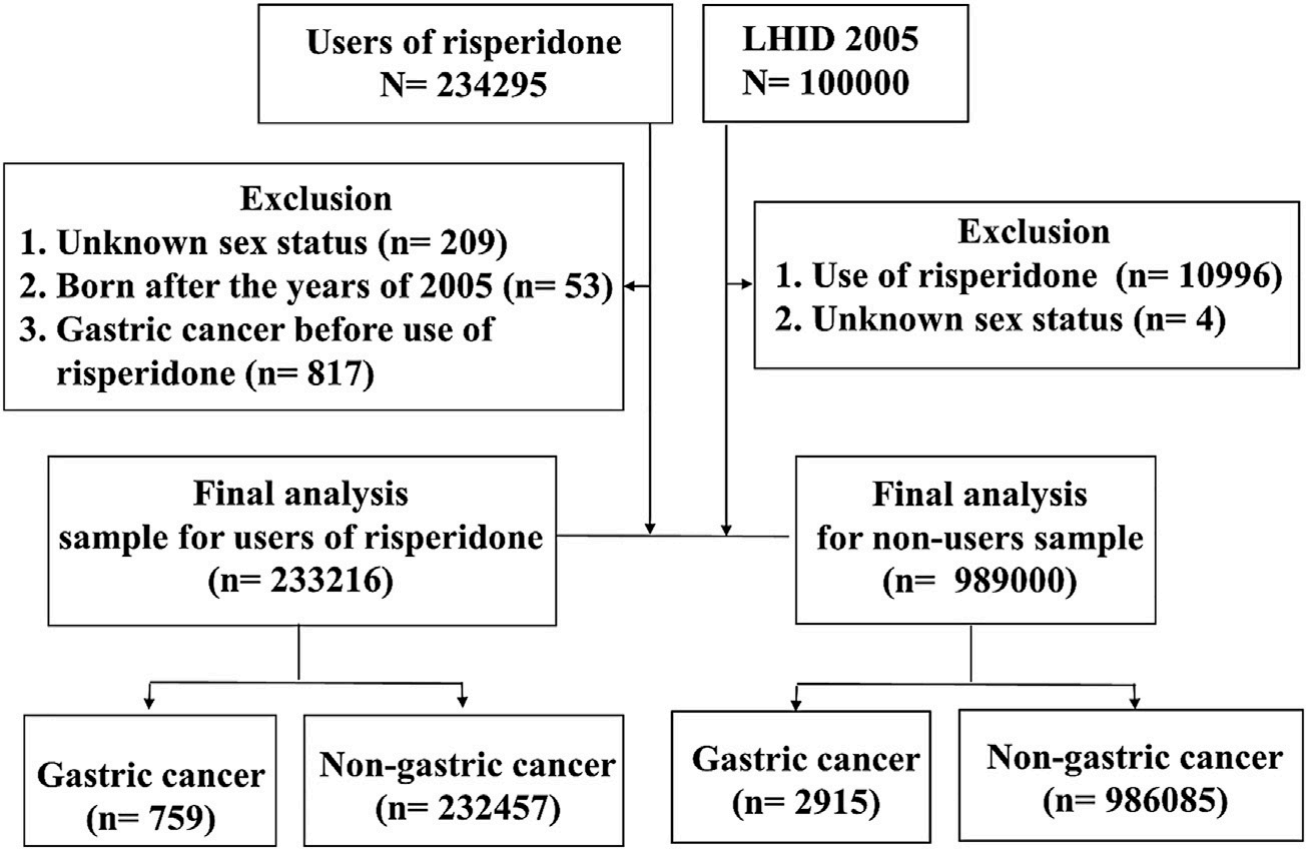
Flow chart of participant selection in the population-based study. LHID, Longitudinal Health Insurance Database.

Figure 5 shows the flow chart of participant selection in the population-based study. Personal and clinical characteristics of our sample are shown in Table 1. Compared with risperidone non-users, risperidone users tended to be older, male and affected by more physical comorbid disorders (i.e., hypertension, hyperlipidemia, diabetes, chronic kidney disease, peptic ulcer, and cirrhosis) and mental comorbid disorders (i.e., major depressive disorder, bipolar disorder, schizophrenia, and alcohol use disorder) with *p* < 0.001. The cumulative incidence of gastric cancer was higher in risperidone users (0.33%) than in nonusers (0.29%), but the mean age of gastric cancer onset was younger in risperidone nonusers than in users (65.3 vs. 73.5 years).

**TABLE 1:** Demographic characteristics of participants in the population-based study.

Variable	User of risperidone *n* = 233,216	Non-users of risperidone *n* = 989,000	*p*-value
	Mean (SD)	Mean (SD)	
Age per year			
Mean (SD)	58.76 (23.46)	43.27 (20.49)	<0.001
Median (IQR)	56 (39–82)	42 (27–57)	
Sex, N (%)			
Female	113,626 (48.72)	498,885 (50.44)	<0.001
Male	119,590 (51.28)	490,115 (49.56)	
Physical comorbid disorder, N (%)			
Hypertension	106,830 (45.81)	216,431 (21.88)	<0.001
Hyperlipidemia	43,529 (18.66)	124,859 (12.62)	<0.001
Diabetes	66,502 (28.52)	122,150 (12.35)	<0.001
Chronic kidney disease	16,526 (7.09)	20,499 (2.07)	<0.001
Peptic ulcer	88,657 (38.01)	223,060 (22.55)	<0.001
Cirrhosis	69,426 (29.77)	191,878 (19.40)	<0.001
Mental comorbid disorder, N (%)			
Major depressive disorder	106,538 (45.68)	65,775 (6.65)	<0.001
Bipolar disorder	46,892 (20.11)	7282 (0.74)	<0.001
Schizophrenia	103,592 (44.42)	4257 (0.43)	<0.001
Alcohol use disorder	7440 (3.19)	1854 (0.19)	<0.001
Drug use, N (%)			
Aspirin	84,880 (36.40)	201,046 (20.33)	<0.001
NSAIDs	224,680 (96.34)	960,876 (97.16)	<0.001
Statins	8671 (3.72)	24,285 (2.46)	<0.001
Outcome, N (%)			
Gastric cancer	759 (0.33)	2915 (0.29)	0.015
Age of onset per year^a^			
Mean (SD)	73.48 (14.98)	65.28 (14.79)	<0.001
Median (IQR)	78 (66–84)	66 (55–77)	

SD, standard deviation; NSAIDs, Non-Steroidal Anti-Inflammatory Drugs; IQR, interquartile range.

a Age of onset of gastric cancer was only estimated in patients with gastric cancer.

#### Association of Risperidone Use With Gastric Cancer Risk

The results of multivariable time-dependent Cox proportional hazard models are presented in Table 2. Negative associations between risperidone use and gastric cancer incidence were observed after controlling for other covariates (HR = 0.75; 95% CI 0.68–0.83 for the one-year induction period and HR = 0.68; 95% CI 0.61–0.75 for the two-year induction period). In the subgroup analysis, similar associations with risperidone use were observed in participants 40 years or older at the index date.

**TABLE 2:** Cox proportional hazard regression model analysis for risk of gastric cancer in the population-based study.

Variable	1-year induction period		2-year induction period	
	Multivariable		Multivariable	
	HR (95% CI)	*p*-value	HR (95% CI)	*p*-value
Risperidone	0.75 (0.68–0.83)	<0.001	0.68 (0.61–0.75)	<0.001
Age per year	1.06 (1.06–1.06)	<0.001	1.06 (1.06–1.06)	<0.001
Male sex	1.49 (1.40–1.60)	<0.001	1.49 (1.40–1.59)	<0.001
Physical comorbid disorder				
Hypertension	0.91 (0.83–0.99)	0.032	0.91 (0.83–0.99)	0.032
Hyperlipidemia	0.99 (0.92–1.08)	0.860	0.99 (0.91–1.07)	0.795
Diabetes	1.03 (0.96–1.11)	0.406	1.03 (0.96–1.11)	0.403
Chronic kidney disease	0.96 (0.85–1.07)	0.473	0.96 (0.86–1.08)	0.482
Peptic ulcer	2.46 (2.29–2.64)	<0.001	2.45 (2.28–2.64)	<0.001
Cirrhosis	1.32 (1.23–1.41)	<0.001	1.31 (1.23–1.41)	<0.001
Mental comorbid disorder				
Major depressive disorder	0.87 (0.80–0.95)	0.002	0.88 (0.81–0.96)	0.003
Bipolar disorder	0.99 (0.83–1.17)	0.889	1.00 (0.84–1.18)	0.959
Schizophrenia	0.83 (0.71–0.96)	0.011	0.85 (0.73–0.99)	0.026
Alcohol use disorder	1.17 (0.83–1.65)	0.366	1.18 (0.84–1.66)	0.346
Drug use				
Aspirin	0.75 (0.69–0.80)	<0.001	0.75 (0.69–0.80)	<0.001
NSAIDs	1.09 (0.95–1.25)	0.239	1.09 (0.94–1.25)	0.261
Statin	0.90 (0.78–1.05)	0.199	0.90 (0.77–1.05)	0.186

NSAIDs, non-Steroidal Anti-Inflammatory Drugs.

## Discussion

This is the first study using a triangulation layout to study the link between risperidone and the hazard of gastric cancer. The present study indicates risperidone potentially exerts anticancer effects, as revealed by the bench study, and the findings can be further replicated in epidemiological studies in a population-based cohort. Several major findings were obtained. First, in the MTT assay, we observed most potential antitumor effect of risperidone compared with other categories of antipsychotics. Second, cell studies showed significant dose-dependent cell viability in Hs27 cells compared with KATO-III cells after risperidone treatment. Second, risperidone inhibited the proliferation of KATO-III cells by inducing apoptosis, and it inhibits the growth of xenograft KATO-III tumors in mouse model. Third, we conducted a population-based study to clarify the relationship between risperidone and gastric cancer epidemically. The result revealed a protective effect of risperidone use (HR = 0.80; 95% CI 0.73–0.88). Consistent results from sensitivity analysis were obtained for the two induction periods (HR = 0.75; 95% CI 0.68–0.83 for one-year and HR = 0.68; 95% CI 0.61–0.75 for the two-year induction period).

Some antipsychotics have been reported to induce autophagy in a variety of cancer cells, and their use is considered a potential strategy for cancer therapy (Vucicevic et al., 2018). Through *in vitro* and *in vivo* experiments, studies have demonstrated that two other antipsychotics, thioridazine (Mu et al., 2014) and sertindole (Dai et al., 2020), possess antigastric cancer effects. These studies have provided no information on real-world applications. Furthermore, the high potential cardiovascular risks of thioridazine (Tisdale, 2016) and sertindole (Leucht et al., 2013) limit their further applications. A study showed that the combined use of tamoxifen and risperidone in patients with breast cancer did not interfere with tamoxifen-induced cytotoxic effects but ameliorated the tamoxifen-induced side effects (Yeh et al., 2014). Moreover, risperidone significantly enhanced the intracellular accumulation of doxorubicin (doxo) in human doxo resistant uterine sarcoma (MES-SA/Dx5) cells, suggesting a beneficial effect of risperidone on overcoming multidrug resistance in cancer therapy (Angelini et al., 2015). These findings also provide evidence of the potential of risperidone for cancer treatment.

Various possible mechanisms of antipsychotic drugs, including risperidone, on preventing cancer cell growth have been emphasized, including inhibition of histone deacetylation (Michaelis et al., 2007), modulation of the dopamine receptor (DR) pathway (Sachlos et al., 2012), and disruption of cholesterol homeostasis (Wiklund et al., 2010). The role of risperidone in the induction of other anticancer mechanisms, such as inhibition of histone deacetylation, modulation of the DR pathway, disruption of the cholesterol homeostasis, or autophagy induction, merits further investigation in clinical practice.

The high effective dose of drugs is frequently a tricky issue in clinics. Similar concern was also found in this study that risperidone exhibits significant cytotoxicity at concentrations higher than 0.1 mM. Interestingly, a recent study reported that rhesus monkeys orally receiving risperidone in a dose-escalation method up to the dose of 0.5 mg/kg/day for 21 days reveal no risperidone-related brain weight changes or gross findings by magnetic resonance imaging (MRI). Although glial fibrillary acidic protein (GFAP) was detected in the brain of these monkeys after 21 days and 12 weeks, no increased GFAP staining was observed after the 12-week recovery period, suggesting this reversible increase of GFAP is likely an adaptive, non-adverse response of astrocytes (Fernandez et al., 2019). In this study, the effective risperidone dose for xenograft tumor experiments is similar to those reported in the study of rhesus monkeys (Fernandez et al., 2019). Therefore, this finding may suggest a possibility that higher-dose of risperidone can be used in treatments of gastric cancer. However, more investigations and safety evaluation should be performed before clinically using risperidone for gastric cancer treatment.

Induction of cell death such as apoptosis, autophagic cell death, necrosis, necroptosis, ferroptosis, entotic cell death and NETotic cell death is currently the primary therapeutic strategy for treating various kinds of cancers (Nonnenmacher et al., 2016; Galluzzi et al., 2018). Accordingly, a recent report indicated that risperidone could cause macroautophagy in a variety of cells and is used as adjuncts in the treatment of schizophrenia, neurodegeneration, and cancers (Vucicevic et al., 2018). Another study also suggested that risperidone can be used for improvement of cancer therapy by inducing cancer cell apoptosis (Hendouei et al., 2019). Similar finding was showed in the current study that risperidone induces apoptosis in gastric cancer cells. Accordingly, these findings suggested that risperidone could exhibit its anti-cancer effect by inducing different ways of cell death. However, the mechanism of action of risperidone in inhibiting gastric cancer is still poorly understood and merits further investigations and clinical trials.

The current study has several limitations. In the bench study, we adopted the xenograft heterotopic model established by subcutaneously injecting cancer cells. This is a low-cost, more reproducible method than orthotopic xenograft models (Vandamme, 2014). However, the orthotopic xenograft models of gastric carcinoma are recommended for simulating a more realistic microenvironment for the development of tumors and validating the treatment (Fidler, 1991). Therefore, appropriate orthotopic xenograft sites are strongly recommended; for example, tumor cells may be injected into the subserosal layer of the stomach (Busuttil et al., 2018) to emulate the microenvironment in which gastric tumors may grow and to confirm the precise mechanism of gastric carcinoma inhibition by risperidone. In terms of the limitation of the population-based study, information on drug adherence was lacking, and data on some major factors related to gastric cancer (e.g., dietary habits) were unavailable in the health insurance claims dataset. The most important strength of the present study was we tried to overcome the above-mentioned limitation by the triangulation study. For example, the *in-vitro* study can be replicated by an *in-vivo* study while the bench study can be confirmed by a real world, population-based cohort study. The persistent findings across different research models validate the beneficial effect of risperidone on gastric cancer.

## Conclusion

**FIGURE 6:**
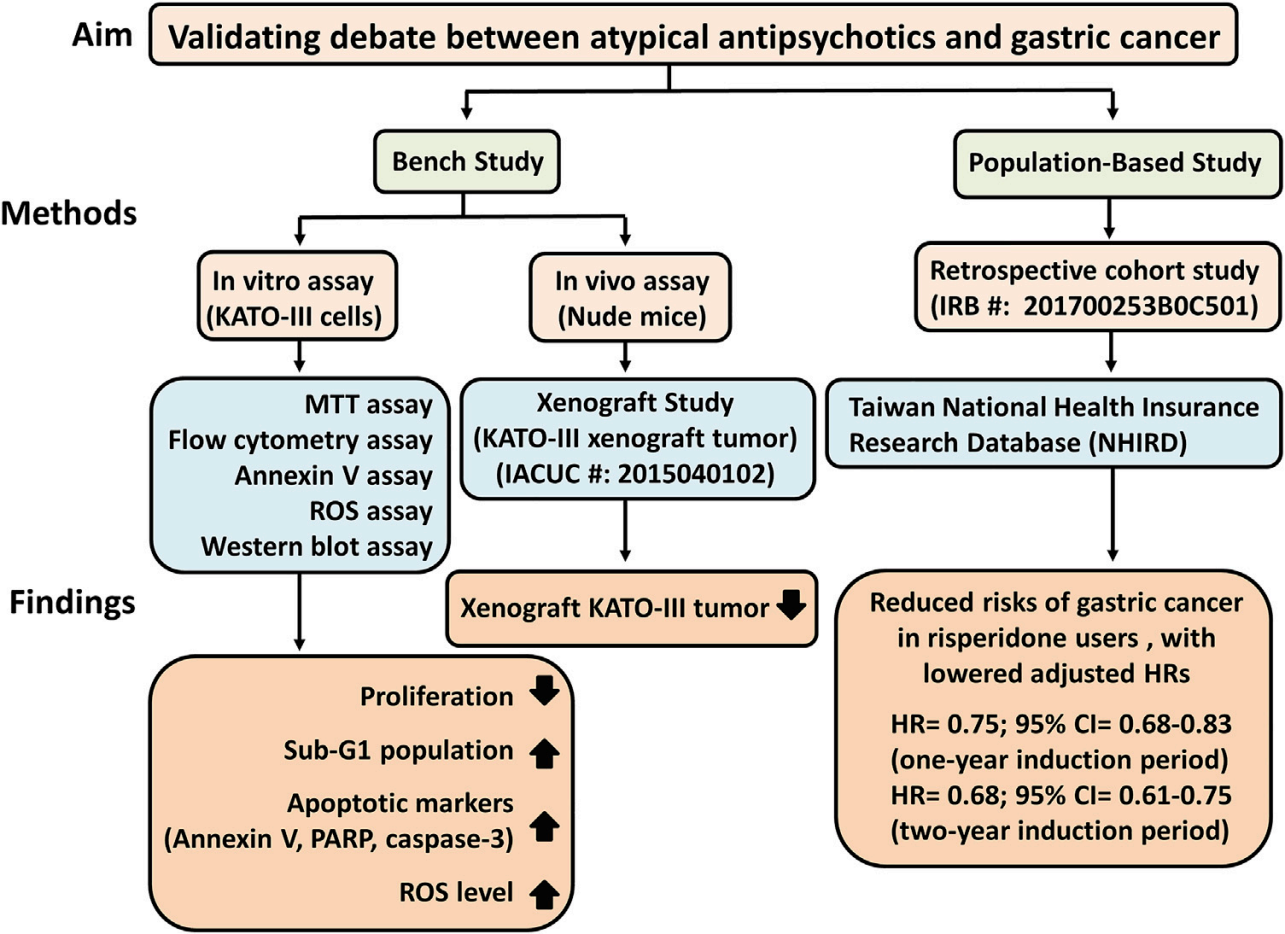
Study schematic diagram of risperidone on gastric cancer. HRs, Lazard Ratios; CI, Confidence Interval.

The major findings of this triangulation study were that risperidone is linked to a lower risk of gastric cancer ( Figure 6 ). We observed significant dose-dependent viability in Hs27 cells in the presence of risperidone compared with that in KATO-III cells. Risperidone inhibited the proliferation of cancer cells through the induction of apoptosis by increased ROS level, and it inhibited the growth of the KATO-III xenograft tumor in mice. The protective effect was also found in real-world population data. Further studies for possible mechanisms and clinical trials are warranted.

## Data Availability

For data of cell study and animal study are included in the article, further inquiries can be directed to the first author and corresponding authors. For data that support the findings cohort study in this study are available from Ministry of Health and Welfare, Taiwan, but restrictions apply to the availability of these data, which were used under license for the current study, and so are not publicly available. Data are however available from the authors upon reasonable request and with permission of Ministry of Health and Welfare, Taiwan.
